# Crosstalk between gut microbiota and metastasis in colorectal cancer: implication of neutrophil extracellular traps

**DOI:** 10.3389/fimmu.2023.1296783

**Published:** 2023-10-23

**Authors:** Jiawei Wu, Wenyan Dong, Yayun Pan, Jingjing Wang, Minliang Wu, Yue Yu

**Affiliations:** ^1^ Department of General Surgery, Changhai Hospital, Naval Medical University, Shanghai, China; ^2^ Clinical Research and Lab Center, Affiliated Kunshan Hospital of Jiangsu University, Kunshan, China; ^3^ Department of Anesthesiology, Changhai Hospital, Naval Medical University, Shanghai, China; ^4^ Department of Burn and Plastic Surgery, Affiliated Kunshan Hospital of Jiangsu University, Kunshan, China; ^5^ Department of Plastic Surgery, Changhai Hospital, Naval Medical University, Shanghai, China

**Keywords:** gut microbiota, colorectal cancer, neutrophil extracellular traps (NET), pre-metastatic niche (PMN), tumor microenvironment

## Abstract

Primary colorectal cancer (CRC) often leads to liver metastasis, possibly due to the formation of pre-metastatic niche (PMN) in liver. Thus, unravelling the key modulator in metastasis is important for the development of clinical therapies. Gut microbiota dysregulation is a key event during CRC progression and metastasis. Numerous studies have elucidated the correlation between specific gut bacteria strains (e.g., *pks* ^+^ *E. coli* and *Bacteroides fragilis*) and CRC initiation, and gut bacteria translocation is commonly witnessed during CRC progression. Gut microbiota shapes tumor microenvironment (TME) through direct contact with immune cells or through its functional metabolites. However, how gut microbiota facilitates CRC metastasis remains controversial. Meanwhile, recent studies identify the dissemination of bacteria from gut lumen to liver, suggesting the role of gut microbiota in shaping tumor PMN. A pro-tumoral PMN is characterized by the infiltration of immunosuppressive cells and increased pro-inflammatory immune responses. Notably, neutrophils form web-like structures known as neutrophil extracellular traps (NETs) both in primary TME and metastatic sites, NETs are involved in cancer progression and metastasis. In this review, we focus on the role of gut microbiota in CRC progression and metastasis, highlight the multiple functions of different immune cell types in TME, especially neutrophils and NETs, discuss the possible mechanisms of gut microbiota in shaping PMN formation, and provide therapeutical indications in clinic.

## 1 Introduction

Colorectal cancer (CRC) is one of the commonest cancers worldwide, with increased CRC incidence in individuals < 50 years of ages, also called young-onset CRC (1). Early stages of CRC patients often receive surgery, although the pre-metastatic niche (PMN) could have already been formed in distal sites, such as liver, lung and lymph nodes. Nearly 25%-30% patients occur liver metastases (2), which is the main cause of death in CRC patients. Moreover, people with inflammatory bowel diseases (IBD) have a strong correlation with tumorigenesis, suggesting that chronic inflammatory environment is a key driving force of CRC (3). Due to the characteristics of gut ecosystem, more and more studies have demonstrated that gut microbiota, including bacteria, fungi, viruses and Archaea are closely related to IBD, CRC and subsequent metastasis. Indeed, gut microbiota can shape host immune system and recruit immunosuppressive cells to booster CRC development; provide genotoxic toxins to cause mutations in colon cells; accelerate the development of CRC by interacting with environmental factors (4). Therefore, unravelling the interplay between gut microbiota and immune system help better understand the pathology and treatment of CRC.

A chronic inflammatory microenvironment is commonly observed in CRC, with the infiltration of different types of immune cells. During innate immune responses, neutrophils serve as first host defense against invading pathogens. Neutrophils kill pathogens through phagocytosis, secretion of toxic granules and most importantly, through the formation of neutrophil extracellular traps (NETs). Upon activation, neutrophils release granule proteins and chromatin and form web-like structures to capture and kill bacteria (5). NETs are firstly identified in 2004 and are thought to be indispensable for bacteria clearance. However, NET has been considered as a double-edge sword in non-infectious diseases, including ischemia reperfusion injury, non-alcoholic steatohepatitis, atherosclerosis and tumors (6–8). NETs can contribute to the progression and metastasis in cancers. Most interestingly, recent studies have demonstrated the existence of NETs in the PMN before the formation of metastases, rendering NET as an important regulator/predictor in CRC progression (9). NETs have also been found in the primary lesion of CRC, while how NETs formed remain largely unexplored. Given that bacteria as one of the most important activators in generating NETs, the crosstalk between gut microbiota and NETs formation has arisen widely attention.

In this review, we discuss gut dysbiosis and the function of immune cells in tumor microenvironment (TME), elucidate how gut microbiota affect PMN formation and metastasis. Moreover, we provide the clinical implications of NETs and gut microbiota in predicting and treating CRC.

## 2 Overview of immune cell landscape and gut dysbiosis in TME

### 2.1 Gut microbiota dysbiosis contribute to the development of CRC

**Figure 1:**
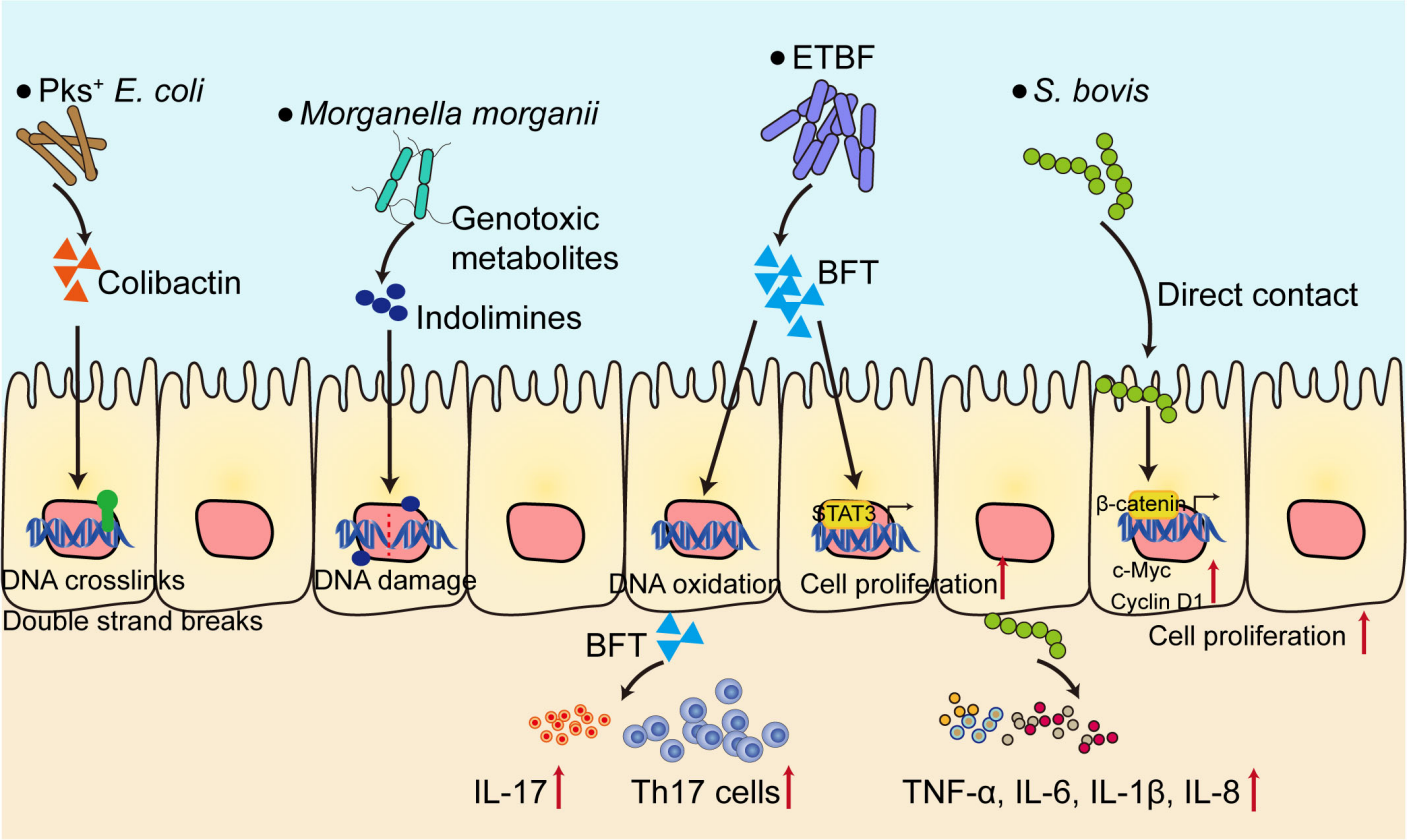
Gut microbiota contribute to the development of CRC. Several bacteria strains (*Pks* ^+^ *E*. *coli*, ETBF, *Morganella morganii*) directly cause DNA damage and genetic alterations through bacteria toxins and metabolites, or activate the transcription factors such as STAT3 and β-catenin, both can promote cell proliferation. On the other hand, bacteria shape the pro-inflammatory microenvironment, characterized by elevated level of pro-inflammatory cytokines (TNF-α, IL-6, IL-1β, IL-8, IL-17), initiate Th17 cell responses to facilitate CRC progression. ETBF, Enterotoxigenic *Bacteroides fragilis*; BFT, *B*. *fragilis* toxin; *S. bovis*, *Streptococcus gallolyticus*; Th17, T helper 17.

One of the most important hallmarks of colorectal tumorigenesis is gut microbiota dysbiosis, characterized by decreased microbial diversity and enrichment of cancer-inducing pathobionts (10). Using metagenomic shotgun sequencing on fecal samples from healthy donors, advanced adenoma and carcinoma patients, a number of *Bacteroides* and *Parabacteroides* species along with *Alistipes putredinis*, *Bilophila wadsworthia*, *Lachnospiraceae bacterium* and *Escherichia coli* were observed in carcinoma and adenoma patients (11). Some bacteria strains have also been shown to be enriched in patients with CRC, including *Bacteroides fragilis*, *pks* ^+^ *Escherichia coli*, *Streptococcus gallolyticus* and *Morganella morganii* (**Figure 1**). These bacteria are closely related to colorectal tumorigenesis (12–15). Enterotoxigenic *Bacteroides fragilis* (ETBF) produces a metalloprotease toxin termed BFT, which has been shown to be closely related to CRC. CRC patients possess higher ETBF colonization when compared to healthy controls (16, 17). Mechanistically, ETBF activates Stat3 in mice and initiates a selective Th17 response, which triggers myeloid-cell-dependent distal colon tumorigenesis (15). ETBF-induced colorectal carcinogenesis is also depended on down-regulating miR-149-3p and promoting PHF5A-mediated RNA alternative splicing of KAT2A in CRC cells (18). ETBF could also increase the stemness of CRC cells *in vitro* and vivo through TLR4-NFAT5-JMJD2B pathway (19). *Pks* ^+^ *Escherichia coli* contain a 50 kb hybrid polyketide-nonribosomal peptide synthase operon (*pks*), which is responsible for the production of genotoxin colibactin (20). *Pks* ^+^ *Escherichia coli* cause interstrand crosslinks and double strand breaks in epithelial cell lines and in mouse models of CRC. The colibactin alkylates DNA and further results in single base substitution (SBS) and small indel signature characterized by single T deletions (21). *Streptococcus gallolyticus* (*S. bovis*) promotes colon cancer progression possibly due to the activation of β-catenin pathway in epithelial cells, further leads to the enhanced expression of c-Myc and Cyclin D1 and promotes cell proliferation (22). Moreover, *S. bovis* stimulates the production of inflammatory cytokines such as TNF-α, IL-6, IL-1β and IL-8 in human colonic cancer cell lines, which establishes a pro-inflammatory microenvironment to facilitate tumor progression (23). *Morganella morganii* produces a family of genotoxic metabolites termed indolimines and induces DNA damage in intestinal epithelial cells which in term causes increased colon tumor burdens in gnotobiotic mouse models (12).

### 2.2 Different immune cells infiltrate TME to influence tumor progression

Immune cells infiltrate tumors while the types of immune responses and their effects on tumor progression and metastasis vary between CRC patients (24). Thus, it is of great importance to understand the complexity of immune cell landscape during the onset of CRC.

#### 2.2.1 Innate immune cells in TME

##### 2.2.1.1 Neutrophils and NETs formation

Neutrophils are most abundant leukocytes in human peripheral blood and serve as important regulators in defense against pathogens. The increased accumulation of neutrophils in both primary tumor sites and metastasis sites are commonly observed in CRC patients (25). Recent studies have shown that tumor-associated neutrophils (TANs) can be polarized to two distinct phenotypes including anti-tumorigenic N1 phenotype and pro-tumorigenic N2 phenotype (26). N1 neutrophils produce cytotoxic cytokines to induce cancer cell death, while N2 neutrophils support tumor growth by expressing arginase, MMP-9, VEGF, CCL2, CCL5 and CXCL4 (27). Notably, most TANs in tumor microenvironment appear to be N2 phenotype, favoring tumor growth and immunosuppression.

Unlike other cells, neutrophils can be stimulated and further form a web-like structure named neutrophil extracellular traps (NETs) through a process called NETosis. NETosis is firstly defined as an important host defense reaction under bacterial infection, since NETs contain anti-bacterial proteins such as histones, neutrophil elastase (NE) accompanied by massive ROS production. NETs are formed in multiple cancer types, including breast cancer, colorectal cancer and lung cancer (28–30). Higher levels of NETs in plasma are found in CRC patients, suggesting that NET can be an important biomarker to predict the development of tumor (31). NETs can support tumor cell metastasis through shielding cancer cells in circulating blood, supporting tumor vasculature outgrowth, promoting cancer-associated thrombosis, regulating epithelial mesenchymal transition and promoting inflammation (32, 33). NETs bind to CCDC25 on tumor cells and subsequently activate ILK-β-parvin pathway to enhance cell motility (34). Tumor-secreted protease cathepsin C promotes breast cancer metastasis by regulating the formation of NETs (30). NE and MMP-9, which are key components in NETs, cleave laminin to induce the proliferation of dormant cancer cells in lung by activating integrin α3β1 signaling (35). Moreover, NETs regulates the efficacy of immunotherapies. In pancreatic cancer, IL-17 drives NETs formation and mediates resistance to immune checkpoint blockade therapy. Inhibition of neutrophils or PAD4-dependent NETosis senses tumor cells to PD-1/CTLA-4 therapy (36). While gut barrier disruption is commonly observed in CRC patients, recent study has pointed that bacteria dissemination from primary CRC could shape PMN in liver. Such metastatic niche is characterized by inflammatory cytokines including TGF-β, CCL2, TNF-α and IL-6. It is noteworthy that our previous data demonstrates neutrophils accumulate in liver before the formation of metastatic lesion, accompanied by NETs formation (37). This provides new insights into NETs in shaping PMN formation in CRC and might in other cancer cell types. Further investigations are needed to clarify the function of early NETosis in distant metastasis lesions.

##### 2.2.1.2 Tumor-associated macrophages

TAMs are recruited by signal molecules including TGF-β, CSF1, CCL2, IL-4 and IL-1. In TME, macrophages can be polarized into two subtypes including classic M1 phenotype and alternative M2 phenotype (38). M1 macrophages are induced by bacterial products and interferons related to type 1 immune responses, while M2 macrophages are induced by cytokines involved in type 2 immune responses such as IL-4 and IL-13 (39). M1 macrophages are associated with tissue damage and tumor cell killing, while M2 macrophages are considered to exert tissue repair and remodeling functions. Indeed, most macrophages in TME are M2-like and the increased number of M2-like TAMs are closely related to the poor prognosis of CRC patients (40). M2 macrophages are considered to be associated with tumor progression and immune suppression. TAMs release CCL5 to inhibit T cell-mediated killing of tumor cells and promote immune escape by stabilizing PD-L1 both *in vitro* and *in vivo* (41). Notably, TAMs could also regulate tumor metastasis, by either regulating epithelial mesenchymal transition process or enhanced angiogenesis (42). SPP1^+^ TAMs exhibit enriched gene signatures involved in Wnt signaling pathway and support tumor growth while CXCL5^+^ TAMs enriched in angiogenesis pathways, indicating that TAMs might promote tumor vasculature to facilitate tumor metastasis (43). Moreover, TAMs also participate in drug resistance via multiple mechanisms. TAMs desensitize CRC cells to 5-fluorouracil treatment via MRP1-dependent drug efflux process by CCL17-CCR4-PI3K-AKT axis in tumor cells (44). Thus, targeting TAMs may provide therapeutical benefits to CRC patients. Until now, several clinical trials have been carried out to reprogram TAMs or functionally inhibit TAMs, including chemokine inhibitors (anti-CCL2 antibody, CCR5 antagonist), CSF1R inhibitors and antibodies, CD47/SIRPα antibodies and CD40 antibodies. The combination of TAMs inhibitors and other drugs including chemotherapeutics and immune-based therapies might be the promising treatment strategy.

##### 2.2.1.3 Dendritic cells

Dendritic cells (DCs) are antigen presenting cells (APCs) and possess the ability to fine-tuning innate and adaptive antitumor immunity. DCs influence tumor progression and clinical outcome of CRC patients (45). The most important function of DCs is antigen presentation. DCs capture antigens and process antigens to form antigenic peptides on MHC I and MHC II molecules, which further lead to the activation of CD4^+^ and CD8^+^ T cells (46). DCs have different subsets including conventional DC (cDC), plasmacytoid DC (pDC) and monocyte-derived DC (moDC). A lower number of colon cancer-infiltrating pDCs is significantly linked to worse prognosis, indicating pDCs are novel prognostic factor in CRC patients (47). Defects in DCs are commonly observed in multiple cancer types, including CRC, which represents a tumor-escape mechanism to generate immunosurveillance (48). Tumor cells secrete cytokines such as VEGF, IL-10, PGE2 and TGF-β to inhibit the function of DCs, characterized by inhibited DC maturation, low MHC II and co-stimulatory molecule expression. For instance, IL-10 blocks the differentiation of DCs from monocytes to impair the APC function of DCs (49), while IL-6 blocks the differentiation of CD34^+^ progenitor cells into DCs and inhibit T cell proliferation (50). Cancer-associated fibroblasts secrete WNT2 to suppress the functions of DCs in TME, and targeting WNT2 restores DC differentiation and enhances the efficacy of immune checkpoints inhibitors (51). Moreover, DCs also express immune checkpoints such as PD-1 and CD80/CD86 molecules on the cell surface to induce T cell exhaustion. Owing to the original function of DCs, numerous studies have pointed that manipulating DCs might be an effective way to treat cancers. DC-based cancer immunotherapy has gained attention over the past few decades. Autologous DCs were isolated from patients and modified ex vivo using patient-specific antigens and re-administrated, to induce intense adaptive immune responses. A variety of antigens have been used to manipulate DCs including CEA, MAGE, HER2 and other tumor-cell derived antigens (whole tumor lysates, DNA, mRNA or whole tumor cells). DC vaccination approach can also serve as an effective treatment against cancers (52). More efforts are needed to clarify specific DC subsets and its functions in TME to establish specific cancer treatments.

#### 2.2.2 Adoptive immune cells in TME

##### 2.2.2.1 T cells

Tumor-infiltrating T lymphocytes are highly heterogeneous within CRC patients and have a large impact on cancer immunotherapies. CRC patients display microsatellite instability (MSI) have better responses to immune-checkpoint blockade of PD-1 than those display microsatellite-stable (MSS), while the underlying mechanisms are not fully understood (53, 54). An integrated approach, named STARTRAC has been carried out to track the dynamic relationship among T cells in CRC and identified 8 CD8^+^ and 12 CD4^+^T cell clusters, including naïve, central memory, effector memory and recently activated effector memory or effector T cells, mucosal-associated invariant T (MAIT) cells, blood-Treg cells, tumor-Treg cells, and exhausted CD8^+^ T cells, Th1-like cells, Th17 cells, follicular T helper and T regulatory cells and tissue-resident memory T cells. Single cell transcriptome analysis reveals exhausted CD8^+^T cells is the most abundant proliferative cells and the transition from effector memory to exhausted T cells mainly occurred in tumor (55). Notably, several mechanisms have been identified to explain CD8^+^T cell exhaustion in TME. Tumoral ammonia levels induce T cell metabolic reprogramming, leading to CD8^+^T cell exhaustion. Enhancing ammonia clearance reactivates T cells, decreases tumor growth, and extends survival in pre-clinical mouse model (56). Matrix Gla protein promotes NF-κB signaling and upregulates PD-L1 expression through enrichment of intracellular free Ca^2+^ levels, thereby facilitates CD8^+^T cell exhaustion (57). High-serum Dickkopf 1 (DKK1) is related to poor response to PD-1 blockade therapy due to the suppression of CD8^+^T cells through GSK3β/E2F1/T-bet axis (54). Thus, restoration of CD8^+^T cell function provide therapeutical benefits in CRC patients.

CD4^+^T cells exhibit lower proliferative signature compared to CD8^+^T cells, with high abundance of tumoral Treg cells in CRC patients. Interestingly, MSI tumors exhibited abundant Th1-like cells, whereas MSS tumors were moderately enriched with Th17 cells. The enrichment of Th1-like cells in MSI patients might contribute to the favorable response to immunotherapies because similar research has demonstrated Th1 cells are involved in anti-CTLA4 therapy in melanoma patients (58). Notably, CRC patients with higher numbers of Th1 cells had prolonged disease-free survival, whereas patients with higher numbers of Th17 cells had a poor prognosis (59). Th17 cells produce Th17-type cytokines (including IL-17A, IL-17F, IL-21 and IL-22), TNF-α and IL-6 to synergistically activate STAT3 and NF-κB to promote cancer cell growth (60). TGF-β signaling in Th17 cells promotes uncontrolled IL-22 release and tumorigenesis in mice through AhR induction and PI3K signaling (61). Treg expansion in tumor microenvironment is also a hallmark of immunosuppression and positively correlated to poor clinical outcomes (62). However, some studies reveal that Treg infiltration correlates with better prognosis (63, 64). This paradoxical phenomenon can be explained by the existence of two types of Treg cells in TME. In CRC patients, infiltrated Treg cells can be sub-divided into two cell types, including FOXP3^lo^ non-suppressive T cells and the suppression competent FOXP3^hi^ Treg cells. CRC patients with abundant infiltration of FOXP3^lo^ T cells showed better prognosis than those with predominantly FOXP3^hi^ Treg cells (65). Thus, depletion of FOXP3^hi^ Treg cells have been proved to augment antitumor immunity and could be used as an effective treatment strategy for CRC.

##### 2.2.2.2 B cells

B cells possess the functions of not only antibodies secretion, but also antigens presentation. B cells have multifaceted roles in tumor progression. The initial studies indicate B cells are mainly pro-tumoral, since the depletion of B cells using monoclonal antibodies showed reduced tumor burden in mice (66). Notably, B cells can generate different immunosuppressive proteins including IL-10, IL-35 and TGF-β, thus inhibit anti-tumor immunity (67). In human CRC, B cell antigen presentation and function are largely attenuated in tertiary lymphoid structures, while B cells in TME might positively correlate to prolonged survival (68). This divergence might due to the heterogeneity and different states of B cells. Using single-cell transcriptome analysis of infiltrating B cell landscape in colorectal cancer, Xia et al. found two main B cell populations including CD20^+^ B cells (mainly localized in tertiary lymphoid structures) and CD138^+^ plasma cells (mainly localized in tumor stroma). Notably, CD20^+^ B cells express high level of immune checkpoint-related genes, which represents the immunosuppressive environment (69). The number of activated B cells in TME are significantly decreased in CRC with liver metastasis, which is correlated to survival and clinical outcomes (70), suggesting that B cells in TME might have anti-tumoral function. However, a leucin-tRNA-synthase-2-expressing B cell subset is found and located outside the tertiary lymphoid structures and correlated with shortened survival (71). Given the close relationship between B cells and tumor prognosis, enhancing the activity of anti-tumor B cell and inhibiting the pro-tumor B cell function can be promising targets.

### 2.3 Interplay between gut microbiota and host immune cells

In CRC progression, gut microbiota can shape host immune responses, while the host immune system can also influence the community of gut microbiota. The infiltration of different immune cells can be directly or indirectly modulated by specific microbes. For instance, *Bacteroides fragilis* initiates the activation of STAT3 and induce a selective Th17 cell infiltration, which contributes to carcinogenesis (15). Gut dysbiosis results in enhanced neutrophil infiltration in abdominal aortic aneurysm (72). Dysregulated microbiota profile stimulates tumor cells to secrete cathepsin K and induce M2 macrophage polarization to enhance CRC metastasis (73). Of interest, metabolites derived from an altered gut microbiome also affect the immune system. Gut microbiota-derived trimethylamine N-oxide, ursodeoxycholic acid and SCFAs modulate the polarization of macrophages (74–76), while butyrate, 3-oxolithocholic acid, and inosine shape T cell immunity (77–79).

Despite the role of gut microbiota in shaping host immune cells, several studies have demonstrated the influence of host immunity on gut microbiota homeostasis. The effector Th 17 cells can regulate the number of bacteria in small intestine, while regulatory T cells suppress the upregulation of IL-23 under bacteria insults (80). Invariant natural killer T cells (iNKT cells) aggravate colonic inflammation by shaping a pro-colitogenic microbiota, *Jα18* ^-/-^ mice lack iNKT cells exihibit elevated abundance of *Rikenellaceae*, *Turicibacteraceae*, *Bifidobacteriaceae* and *Prevotellaceae* (81). The offspring of *Tcrδ* ^-/-^ mice harbors a distinct intestinal microbiota and decreased level of intestinal SCFAs, further sensitizes mice to first-breath-induced inflammation, unravelling a maternal γδT cell-microbiota-SCFA axis in regulating lung inflammation (82). Since the intertwined fates between gut microbiota and host immunity, the cause link between gut microbiota and immune response remains further investigation.

## 3 Gut dysbiosis in facilitating NETs formation and metastasis

### 3.1 Mechanisms of NETs formation

**Figure 2:**
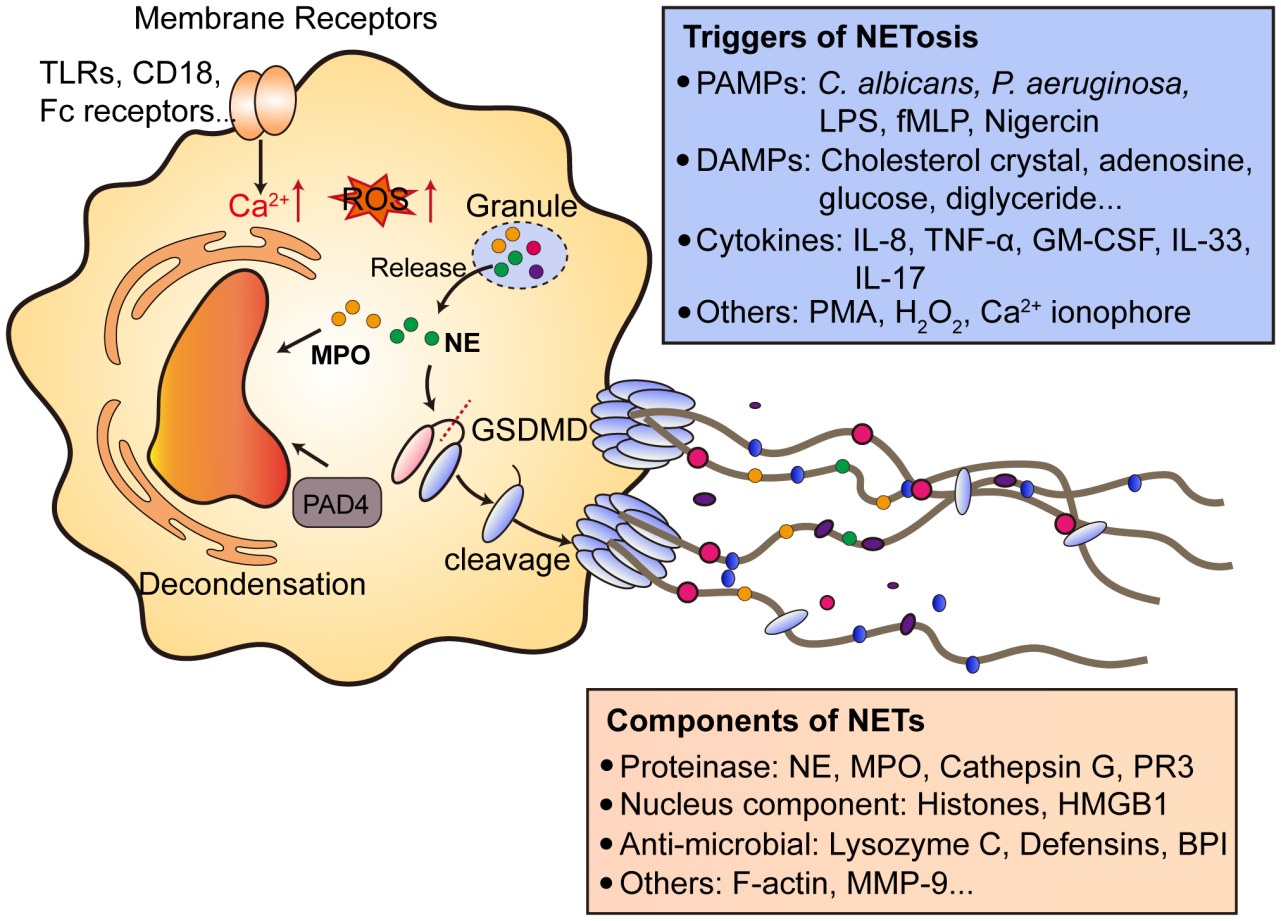
Mechanisms of NETs formation. After the activation of neutrophils by various cell membrane receptors signaling, invading pathogens and DAMPs, which causes the downstream Ca^2+^ release from endoplasmic reticulum and ROS production. The release of granule proteins such as NE and MPO facilitates the decondensation of chromatin accompanied by PAD4. PAD4 citrullinates histones to release DNA. Notably, NE processes GSDMD to generate N-terminal GSDMD and form pores on plasma membrane to help NETs extrusion. NETs contain proteases, nucleus components, anti-microbial proteins and other proteins like F-actin and MMP9. NETs can be induced by multiple factors, including PMAPs (bacteria, LPS), DAMPs and cytokines. NETs formation can also be triggered by PMA, which activates PKC and promotes ROS production. TLRs, toll-like receptors; Fc, antibody fragment; ROS, reactive oxygen species; MPO, myeloperoxidase; NE, neutrophil elastase; PAD4, protein arginine deaminase type 4; GSDMD, gasdermin D; PAMPs, pathogen-associated molecular patterns; LPS, lipopolysaccharides; fMLP, N-formylmethionyl-leucyl-phenylalanine; DAMPs, danger-associated molecular patterns; NETs, neutrophil extracellular traps; PR3, proteinase 3; HMGB1, high mobility group protein B1; BPI, bactericidal permeability-increasing protein; MMP-9, Matrix metalloproteinase-9. PMA, phorbol 12-myristate 13-acetate; PKC, protein kinase C. GM-CSF, granulocyte-macrophage colony-stimulating factor.

NETs consist of decondensed chromatin and form web-like DNA structures with nuclear proteins, granule proteins and cytosolic proteins. Until now, the clear mechanism of NETs formation and NETosis remains poorly understood. Basically, two types of NETosis are found, including lytic NETs formation (slow cell death) and alternative non-lytic NETs formation (independent of cell death and rapid release of nuclear chromatin accompanied by granule proteins) (83). To initiate NETosis, neutrophil activation is a prerequisite, as resting neutrophils do not undergo NETosis. Neutrophil activation requires membrane surface receptors (such as Toll-like receptors, CD18 and Fc receptors) or bacterial toxins, which finally leads to an increase of intracellular calcium concentration (84–87). Then the cell-extracellular matrix adhesion and plasma membrane microvesicles shedding increased, accompanied by actin disassembly and vimentin remodeling. Next, the chromatin decondensation occurred, which is most likely induced by histone citrullination. Citrullinated histones induce chromatin decondensation by decreasing the electrostatic interaction between histone and DNA. This process is mainly supported by protein arginine deaminase (PAD) family proteins (88, 89). Notably, protein arginine deaminase type 4 (PAD4) is mainly expressed in granulocytes. PAD4 causes the citrullination of histone H3 at arginines 2, 8, 17 and 26 and H4 and H2A at arginine 3 (90, 91). Due to its function, PAD4 has long been considered as a key player in chromatin decondensation during NETosis. Neutrophils from *Pad4*-KO mice do not undergo NETosis induced by PMA and LPS (92). Pharmacological inhibition of PAD4 could also diminishes NETosis and subsequent NETs release (93, 94). However, some studies pointed that PAD4 may not be involved in NETosis under certain stimulus, since *C.albicans* also induce NETosis in *Pad4*-KO mice (95). Besides, when neutrophils undergo NETosis, granule proteases such as NE and PR3 could also induce chromatin decondensation, possibly via histone cleavage (96, 97). This process needs the translocation of proteases from granules to cytosol, and further into nucleus. After DNA decondensation, DNA escape from the nucleus with the help of lamin remodeling and nuclear envelope holes formation. Finally, DNA break through the plasma membrane and is released extracellularly (**Figure 2**). Lytic NETosis is often accompanied by the rupture of plasma membrane. Of note, recent studies have pointed out the significant role of pore-forming proteins in NETosis, especially Gasdermin D (GSDMD). GSDMD plays an important role in the generation of neutrophil extracellular traps (98). GSDMD is cleaved by inflammatory caspases (Caspase-1/4) to generate N-terminal GSDMD and located on plasma membrane to form pores of about 20 nm in diameter (99). Interestingly, N-GSDMD is not only processed by caspases, as NE also cleaves GSDMD at C268 (100). The cleaved GSDMD by NE also have the ability to form pores on cell membrane. Notably, the active form of GSDMD is not only located on cell membrane, but also other organelles, such as mitochondria and nucleus (101). Whether active GSDMD facilitates proteases release from granules and form a positive feed-back loop remain further investigation.

Neutrophils can be triggered to undergo NETosis through a lot of stimuli, including (1): bacteria and bacteria components like LPS, fMLP and Nigercin (2); cytokines like IL-8, TNF-α, GM-CSF, IL-33 and IL-17 (3); others like PMA, H_2_O_2_ and Ca^2+^ ionophore. Of note, endogenous metabolites can be neglected causes of NETosis, especially in non-infectious diseases. Our previous research demonstrated linoleic acid is an important driving force of NETs formation in NASH, which promotes uncontrolled inflammation during disease onset (7). Patients with mutations in ADA2 gene results in a systemic vasculitis accompanied by elevated adenosine level. Adenosine triggers NETs formation and exacerbate disease progression (102). High glucose level is also considered to trigger NETosis in diabetic patients and impair wound healing (103). Cholesterol causes NETosis and primes macrophages for production of inflammatory cytokines to amplify immune responses during atherosclerosis (104). The driving force of NETosis in CRC remain poorly understood, current opinions mostly believe that cytokines or proteins secreted by tumor cells are initiators of NETosis. In CRC, no metabolic factors have been identified as NETosis triggers, while the dysregulated cellular metabolism is a key hallmark of colorectal cancer (105). Thus, further studies might focus on the metabolic regulation of NETs formation in both primary lesions or metastatic sites.

### 3.2 Gut microbiota in shaping PMN: potential role of NETs formation

#### 3.2.1 Gut microbiota translocation is a key event during CRC progression

Under normal condition, the intestinal barrier is a physical and chemical barrier that separate host apart from pathogens, invasive bacteria and viruses. Mucosal layer and immune system are the chemical barriers in intestine, the mucus layer also contains the commensal bacteria and produces antimicrobial proteins and secretory IgA. The intestinal epithelial cell layer acts as the first physical barrier, and its integrity is maintained by tight junction proteins, including occludin, claudins and zonula occludens (106). The loss of epithelial integrity is commonly witnessed in CRC patients and animal models. The translocation of several bacteria strains including *E. coli* and *Enterococcus faecalis* have been proved and thus promotes the activation of innate and adaptive immune cells to exacerbate inflammation at primary sites. The elevated inflammation in intestine may further disturb the intestinal barrier function and damage the barrier, which in turn causes more bacteria translocation. Notably, some commensal bacteria may acquire the virulence factors and become pathogenic, thus fueling CRC progression (107). In addition to mucus and intestinal barrier, the gut is also equipped with gut vascular barrier, and serves as a gatekeeper to limit the access of microorganisms into blood circulation (108). The disruption of gut vascular barrier may facilitate bacteria and even cancer cells translocation into blood. Impaired intestinal barrier leads to bacteria dissemination into tumor sites and further modify the gut vascular barrier, migrate to the liver to form a favorable PMN for subsequent CRC seeding. The translocation of bacteria from lumen to metastatic lesion fosters neutrophils recruitment in liver and creates pro-inflammatory immune microenvironment (109). Thus, the translocation of bacteria not only promotes the primary site tumor cell growth, but also metastasis.

#### 3.2.2 Participators in PMN formation

Primary CRC can shape liver microenvironment form a favorable PMN to facilitate metastasis, possibly by mediating immune cells recruitment, immunosuppression, vascular leakage and inflammation (110). Chemokines secreted by either tumor cells or cells reside in TME have impacts on PMN, including CXCL1, CCL2, CCL9 and CCL15. CXCL1 interacts its receptor CXCR2 on numerous immune cells and regulates the migration and recruitment of cells. It is reported that CXCL1 is important for PMN formation by recruiting CXCR2-positve myeloid-derived suppressive cells (MDSCs) (111). CCL2 enhances the infiltration of macrophages in liver and regulates it polarization to a M2 phenotype (112). Thus, the recruitments of different immune cells triggered by chemokines are key events in PMN formation. Bone marrow-derived cells, especially MDSCs, neutrophils and macrophages are important for PMN formation. MDSCs are firstly found in cancer patients with strong immunosuppressive activity, which can be divided into granulocytic MDSCs (G-MDSCs) and monocytic MDSCs (M-MDSCs). MDSCs could suppress T cell activity to help tumor cells escape immune surveillance, inducing tumor cell invasion and proliferation and induce angiogenesis (113).

In addition to MDSCs, pro-tumoral N2 neutrophils release LCN2 and further boost mesenchymal-epithelial transition of circulating tumor cells (114). Depleting neutrophils using Ly6G neutralizing antibody causes a remarkable reduction of metastasis without affecting the metastatic potential of primary tumors (115). Recently, NETs are considered to be involved in PMN formation. NETs occurred in lymph nodes positively correlates to reduced patient survival, and promotes metastasis, while blocking the NETs formation could efficiently suppress lymph node metastasis (116). In early-stage ovarian cancer, NETs are formed and detected in omentum before metastasis, NETs help shield tumor cells in blood and facilitate metastasis (9). Sustained lung inflammation induces NETs formation and awaken the dormant cancer cells in lung. NE and MMP9 cleave laminin to induce the proliferation of dormant cancer cells (35). NETs are mainly formed in PMN by cytokines like CXCL8, complement C3 and IL-1β (117). Notably, Zeng et al. reported oxalate accumulation in PMN induced NETs formation due to the upregulation of hydroxyacid oxidase 1 in alveolar epithelial cells, suggesting a non-negligible role for metabolic reprogramming in NETs formation (118). Further studies are needed to investigate the mechanisms of NETs formation in pre-metastatic sites.

Moreover, cancer-derived exosomes are important players in PMN formation. In CRC, miR-25-3p are transferred from CRC cells to endothelial cells via exosomes and further regulates the expression of tight junction proteins in ECs, thus promotes vascular permeability (119). CRC-derived exosomal miR-203a-3p induce M2 polarization of macrophages and increase the metastatic potential of CRC (120). Exosome-derived HSPC111 also promotes PMN formation and metastasis by reprograming lipid metabolism (121). Exosome-derived ADAM17 is upregulated in CRC patients and promotes the migratory ability of CRC by cleaving E-cadherin junction (122).

#### 3.2.3 Gut microbiota: new aspects of PMN formation

Given the dysregulated microbiota profiles during CRC progression, and bacteria translocation is commonly observed, gut microbiota may be involved in PMN formation. Our previous data supported the notion that long-term, high-concentration of capsaicin in diet could shape liver PMN prior to CRC metastasis, along with bacteria translocation to liver (37). Moreover, microbial disturbance caused by diet may have an impact on bacteria translocation and PMN formation. High-fat diet promotes lung PMN formation and metastasis through changes of microbiota (123). Recently, the tumor-resident bacteria *E.coli* found in CRC are considered to disrupt gut vascular barrier. The disruption of barrier causes the dissemination of bacteria to liver, fostering the formation of PMN (109). The gut-liver axis is important in bacteria dissemination and thus might explain why CRC often leads to liver metastasis. Gut bacteria initiate a pro-inflammatory response in liver by upregulating the expression of chemoattractants such as *Saa1/2/3/4*, *Lcn2* and *Tlr5*, thereby enhance the PMN formation. However, the deeper mechanistic studies are needed to unravel how gut microbiota initiates pro-inflammatory responses and key microbiota-immune cell relationships in liver. In CRC patients, gut microbiota might be the initiator of liver PMN formation.

Notably, since how NETs formed in metastatic niche remain uncertain, it is possible that in CRC, gut microbiota is an important mechanism in regulating NETs formation in liver. NETosis can be triggered by either bacteria or bacteria components (**Figure 2**). Studies have proved that gut bacteria can regulate NETs formation. In abdominal aortic aneurysm patients, gut microbiota dysbiosis leads to increased neutrophil infiltration and NETs formation. Metabolomics analysis revealed *R. intestinalis*-derived metabolite butyric acid could inhibit NETs formation *in vivo* by regulating NOX2 expression (72). Similarly, microbiota-derived butyrate also inhibits NETs formation and inflammatory cytokines secretion in IBD patients (124). In acute mesenteric ischemia-reperfusion injury, antibiotic-treated mice or germ-free mice showed increased NETosis, further suggesting the potential role of gut microbiota in NETs formation (125).

## 4 Clinical applications of NETs and gut microbiota in CRC

### 4.1 Gut microbiota as a biomarker in CRC

Due to the significance of some specific bacteria strains in CRC progression, gut microbiota biomarkers have potential translational applications in CRC screening and early diagnosis. Faecal immunochemical testing (FIT) is often used to screen CRC, which lacks the sensitivity, especially for adenomas (126). Thus, large-scale assessments of gut microbiome between CRC patients and healthy people have been carried out and some bacteria strains are able to discriminate between patients and those without cancer with a high level of accuracy (area under curve AUC > 0.9). *Fusobacterium nucleatum* (*Fn*) was reported to be more abundant in colorectal cancer than controls, with AUC of 0.868. The abundance of *Fn* could also discriminated colorectal cancer from controls with a sensitivity of 77.7%, which provide valuable diagnostic biomarkers for clinical colorectal cancer (127, 128). The increased abundance of *Clostridium symbiosum* was found in colorectal adenoma, early CRC and advanced CRC patients, being a promising biomarker for early and noninvasive detection of CRC. Notably, when combining *Clostridium symbiosum* and FIT or CEA, the diagnosis power is improved. A signature combining *Fn*, *C. hathewayi*, *Bacteroides clarus* and m7 resulted in a superior AUC of 0.89 (129). Beyond bacteria, multi-kingdom microbiota analyses revealed 16 multi-kingdom markers including 11 bacterial, 4 fungal and 1 archaeal feature and showed good performance in diagnosing patients with CRC (AUC=0.83) (130), suggesting that when using microbiota as diagnosis markers, it is better to combine more markers due to the complexity of gut microbiota composition.

### 4.2 NETs as an indispensable biomarker in CRC metastasis detection

NETs are easily and efficiently detected in patient serum samples by analyzing the MPO-DNA complex. Given the pro-tumoral effects of NETs in cancers, PMN formation and subsequent cancer metastasis, NET becomes a promising biomarker to predict the metastasis potential. Elevated NETs formation is strongly correlated to a higher risk of metastasis. In patients with breast cancer, higher levels of serum NETs are associated with subsequent metastasis to liver (34). In a cohort of 85 patients with CRC, NETs are significantly associated with lymph node metastasis (131). Thus, analyzing the NETs in peripheral blood offers a new way to predict the metastasis potential in cancer patients. Moreover, some studies pointed out that NETs also correlate with survival of cancer patients. The infiltration of NET-release neutrophils is negatively associated with the survival of HCC patients (132). The level of NETs is an independent prognostic factor for progression-free survival in patients with advanced gastric cancer (133). A NET-associated gene signature is also efficient to predict the overall survival in gastric cancer (134).

### 4.3 Pharmacological inhibition of NETs formation

Since NETs formation is important in both primary CRC and PMN formation, targeting NETosis and NETs may provide therapeutical benefits to patients. Unlike animals, neutrophils cannot be depleted using neutralizing antibodies due to its important role in host defense against pathogens in humans, but several pharmacological approaches have achieved successful benefits both in pre-clinic and clinic. So far, two methods have been used to inhibit NETosis, including degradation of NETs using DNase I and inhibition of PAD4 using small-molecule inhibitors. While DNase I could cause impaired host defense against infection, tumor specific delivery and release of DNase I in tumor sites breaks NET-mediated physical barrier and increases the contact of cytotoxic T cells and sensitizes immune checkpoint blockade therapy in CRC (135). Biomimetic CCDC25-overexpressing cell membrane hybrid liposomes loaded with DNase I efficiently eliminates NETs and inhibits CRC liver metastasis (136). PAD4 inhibitors include Cl-amindine, GSK484, YW3-56 and some other peptide-based inhibitors. However, due to the highly conserved structure of all PAD isozyme active sites, it is difficult to design and develop selective PAD4 inhibitors, most of the inhibitors are pan-PAD inhibitors (137). Cl-amidine covalently modifies the active site cystine of PAD4 and irreversibly inactivate PAD4 in a calcium-bound state (138). Cl-amidine could efficiently inhibit histone H3 citrullination and NETs formation both *in vitro* and *in vivo* (93). Due to the poor cell permeability of most PAD inhibitors, YW3-56 is developed to achieve better cell permeability with an improved inhibitory effect on PAD4 enzymatic activity (139). The development of high selective, improved bioavailability PAD4 inhibitors are still needed in future.

### 4.4 Manipulating of the gut microbiota

The dysregulated microbiota in CRC patients prompts us to recover the homeostasis of gut microbiota. Fecal microbiota transplantation (FMT) is often used to alter the gut microbiota composition, and has been designated as a biological drug by FDA (140). In *Clostridium difficile* infection (CDI), FMT is used in clinical settings and the effectiveness of FMT for clinical cure of recurrent CDI approximately 90% (141). By transferring the microbiota from healthy mice to CRC mice, cancer progression is significantly inhibited, while microbiota from patients with CRC can drive carcinogenesis in both germ-free mice and mice received carcinogen azoxymethane, suggesting the potential role of FMT in CRC treatment (142, 143). Moreover, FMT also improves immunotherapies and chemotherapy responses. The intact commensal bacteria community controls the response of chemotherapy in CRC (144). In melanoma patients, recent clinical trials demonstrate FMT can overcome the resistance to anti-PD-1 therapy and provide therapeutical benefits in patients (145, 146). *Bacteroides fragilis* and *Bacteroides thetaiotaomicron* govern the efficacy of CTLA blockade therapy in tumor patients, since FMT from human to mice confirms the positive correlation between *B.fragilis* and CTLA-4 responses (147).

Using antibiotic to inhibit cancer-associated bacteria like *Fusibacterium* reduces cancer cell proliferation and overall tumor growth in *Fusobacterium*-associated colorectal cancer (148). In addition, colonization of several commensals can enhance cancer immune therapies. Oral supplementation with *Akkermansia muciniphila* could sensitize patients to PD-1 inhibitors (149). Colonization of *Bifidobacterium* improves the efficacy of PD-L1 checkpoint blockade therapy and leads to CD8^+^T cell accumulation in TME (150). More studies are needed to bring pre-clinical investigations into clinical trials and provide real benefits to CRC patients.

## 5 Conclusion

In this review, we summarize the recent advances of gut dysbiosis and immune cells in CRC progression and metastasis, highlight the role of NETs in facilitating CRC metastasis. Notably, gut microbiota translocation may be a potent regulator of PMN, possibly by regulating of the formation of NETs. Thus, a deeper understanding of the gut bacteria dysbiosis during CRC progression, along with the interplay between gut bacteria and immune cells not only in TME, but also in PMN may provide the thorough development of anti-tumor therapies in future. So far, it is of great interest that FMT could serve as a standardized treatment of gut dysbiosis in multiple diseases. While the definition of healthy microbiota remains further investigation, the FMT process may have side-effects in the context of clinical treatments. Thus, a personalized gut microbiota analysis is needed to achieve precise editing of gut microbiome to combat CRC progression and metastasis. In addition, both NETs and gut microbiota can be useful clinical indicators to predict the progression and metastasis potential of primary CRC, but have no unified conclusions till now. The combination of both might provides more accurate prediction in clinical settings.

## Author contributions

JWW: Conceptualization, Data curation, Formal Analysis, Investigation, Methodology, Resources, Software, Supervision, Visualization, Writing – original draft, Writing – review & editing. WD: Conceptualization, Formal Analysis, Methodology, Software, Writing – review & editing. YP: Conceptualization, Investigation, Resources, Software, Writing – review & editing. JJW: Conceptualization, Investigation, Methodology, Supervision, Writing – review & editing. MW: Formal Analysis, Investigation, Methodology, Supervision, Writing – review & editing. YY: Conceptualization, Funding acquisition, Project administration, Supervision, Writing – original draft, Writing – review & editing, Investigation, Methodology, Resources, Validation, Visualization.
